# Ginsenoside Rg1 for ischemic heart disease injury: preclinical evidence and possible mechanisms

**DOI:** 10.1016/j.jgr.2026.101126

**Published:** 2026-08-17

**Authors:** Jiaxin Chen, Sanjian Hu, Yujie Zhang, Ziyou Liu, Yuting Huang

**Affiliations:** a Key Laboratory of Prevention and Treatment of Cardiovascular and Cerebrovascular Diseases of Ministry of Education, First Affiliated Hospital of Gannan Medical University, Gannan Medical University, Ganzhou, 341099, China; b School of Pharmacy, Gannan Medical University, Ganzhou, 341000, China; c School of the First Clinical Medicine, Gannan Medical University, Ganzhou, 341000, China

**Keywords:** Cardioprotection, Ischemic heart disease, Ginsenoside Rg1

## Abstract

**Background:**

Ischemic heart disease (IHD) is the leading cause of global morbidity and mortality, underscoring an urgent need for innovative therapeutic strategies. *Panax ginseng C. A. Meyer*, a cornerstone herb in traditional medicine with exceptional pharmacological value, is widely regarded as the "King of Herbs". Ginsenoside Rg1 (G-Rg1), one of the principal bioactive protopanaxatriol-type saponins isolated from *Panax ginseng C. A. Meyer*, exhibits well-documented cardioprotective effects. However, a systematic and quantitative synthesis of its efficacy and mechanisms in preclinical IHD models is still lacking.

**Methods:**

In this study, we conducted a preclinical systematic review to evaluate the efficacy of G-Rg1 in animal models of IHD and to synthesize current evidence on its underlying mechanisms. A search of four major biomedical databases from inception to May 2026 identified nine eligible studies (236 animals). Methodological quality, rigorously assessed using the 9-item CAMARADES checklist, yielded scores of 4 to 6, indicating moderate overall quality. Data were pooled and analyzed using RevMan 5.3.

**Results:**

Meta-analysis showed that G-Rg1 administration reduced myocardial infarct size and decreased plasma levels of cardiac injury biomarkers (creatine kinase-MB [CK-MB] and N-terminal pro-brain natriuretic peptide [NT-proBNP]), and improved key functional parameters. One study reported that G-Rg1 attenuated the inducibility of ventricular tachyarrhythmias following ischemia-reperfusion injury. Mechanistically, the cardioprotective effects of G-Rg1 were consistently associated with anti-inflammatory, anti-apoptotic and mitochondrial protection pathways.

**Conclusion:**

These preclinical findings support future translational research and provide a basis for subsequent clinical studies on G-Rg1 in IHD.

## 1 Introduction

In recent years, the global prevalence of metabolic risk factors, including hypertension, dyslipidemia, and obesity, has risen markedly, driven by sedentary lifestyles and an aging population [1]. These factors accelerate the progression from isolated metabolic disturbances to metabolic syndrome, a cluster of abnormalities that substantially elevates the risk of multiple chronic diseases, particularly cardiovascular disease (CVD) [2]. CVD remains the leading cause of death worldwide, accounting for more deaths annual than any other disease, and is projected to claim approximately 23.6 million lives by 2030 [3]. Among CVD subtypes, IHD, which encompasses acute myocardial infarction (MI), chronic myocardial atrophy, and consequent heart failure [4,5], imposes the greatest global disease burden and mortality [2,6]. IHD-induced myocardial injury stems from a cascade of interrelated pathological processes, including ischemia, hypoxia, inflammation, oxidative stress, and calcium overload, that collectively drive cardiomyocyte necrosis, impair reparative responses, disrupt myocardial architecture, compromise systolic and diastolic function, and trigger maladaptive ventricular remodeling, ultimately threatening both survival and quality of life [[7], [8], [9], [10]]. Widely used experimental models of IHD-related injury include myocardial ischemia/reperfusion (I/R) [11] and permanent coronary artery ligation to induce MI [12]. Both models faithfully recapitulate key features of human IHD, such as irreversible cardiomyocyte loss and deficient tissue repair, leading to progressive ventricular dysfunction and heart failure. Consequently, the development of novel, mechanism-based cardioprotective strategies to mitigate myocardial injury and preserve cardiac function remains a pressing priority in cardiovascular medicine.

*Panax ginseng C. A. Meyer*, a perennial herb of the Araliaceae family, is one of the most revered medicinal plants and is widely acclaimed as the "King of Herbs". Its therapeutic use dates back over two millennia, with documented applications in the Divine Farmer's Materia Medica, where it was prescribed for vital energy (Qi) replenishment, fatigue alleviation and organ system regulation. Modern pharmacological research confirms that ginseng exerts pleiotropic effects across multiple physiological systems, including the nervous, cardiovascular, endocrine, gastrointestinal, reproductive and respiratory systems [13], largely attributable to its diverse array of bioactive constituents, particularly the triterpenoid saponins known as ginsenosides [14]. Recent developments (2025 to 2026) have further propelled the field of ginsenoside cardiovascular research. A study in a mouse MI model demonstrated that biodegradable mesoporous basic copper carbonate microspheres loaded with ginsenoside Rb1 (G-Rb1), significantly improved cardiac function and promoted vascularization in myocardial tissue, highlighting the beneficial effects of combining drugs with bioactive carriers for myocardial regeneration [15]. Concurrently, lipid-based encapsulation combined with pulmonary perfusion administration significantly enhanced the bioavailability of G-Rg1 in rats, renewing interest in this compound, which was previously limited by poor bioavailability [16]. Moreover, single-cell transcriptomic analysis revealed that G-Rg1 modulates the NDUFS4-SIRT5-DUSP1 axis, regulates the mitochondrial quality control network, and attenuates the inflammatory cascade in coronary microvascular endothelial cells from mice subjected to ischemia-reperfusion injury, offering deeper mechanistic insight [17]. These advances highlight the importance of a timely synthesis of preclinical evidence on G-Rg1 in ischemic myocardial injury.

G-Rg1 is a protopanaxatriol-type ginsenoside isolated from the root of *Panax ginseng C. A. Meyer*. It is among the abundant and pharmacologically active compounds, and its chemical structure is illustrated in Fig. 1. Accumulating preclinical evidence highlights G-Rg1's cardioprotective properties [18,19], including attenuation of cardiomyocyte oxidative stress injury [20], enhancement of mitochondrial biogenesis and ATP synthesis [21], suppression of NF-κB-mediated proinflammatory signaling [22], and modulation of calcium handling and autophagic flux. Despite these promising findings, no systematic synthesis has yet evaluated the overall efficacy, optimal dosing range, or mechanistic consistency of G-Rg1 across experimental models of myocardial injury. Therefore, based on published animal experiment literature, this study conducted a systematic review and meta-analysis to clarify the protective effect of G-Rg1 on myocardial injury and its potential mechanism, providing reliable evidence-based medical evidence for its subsequent basic research and clinical translation.

**Fig. 1:**
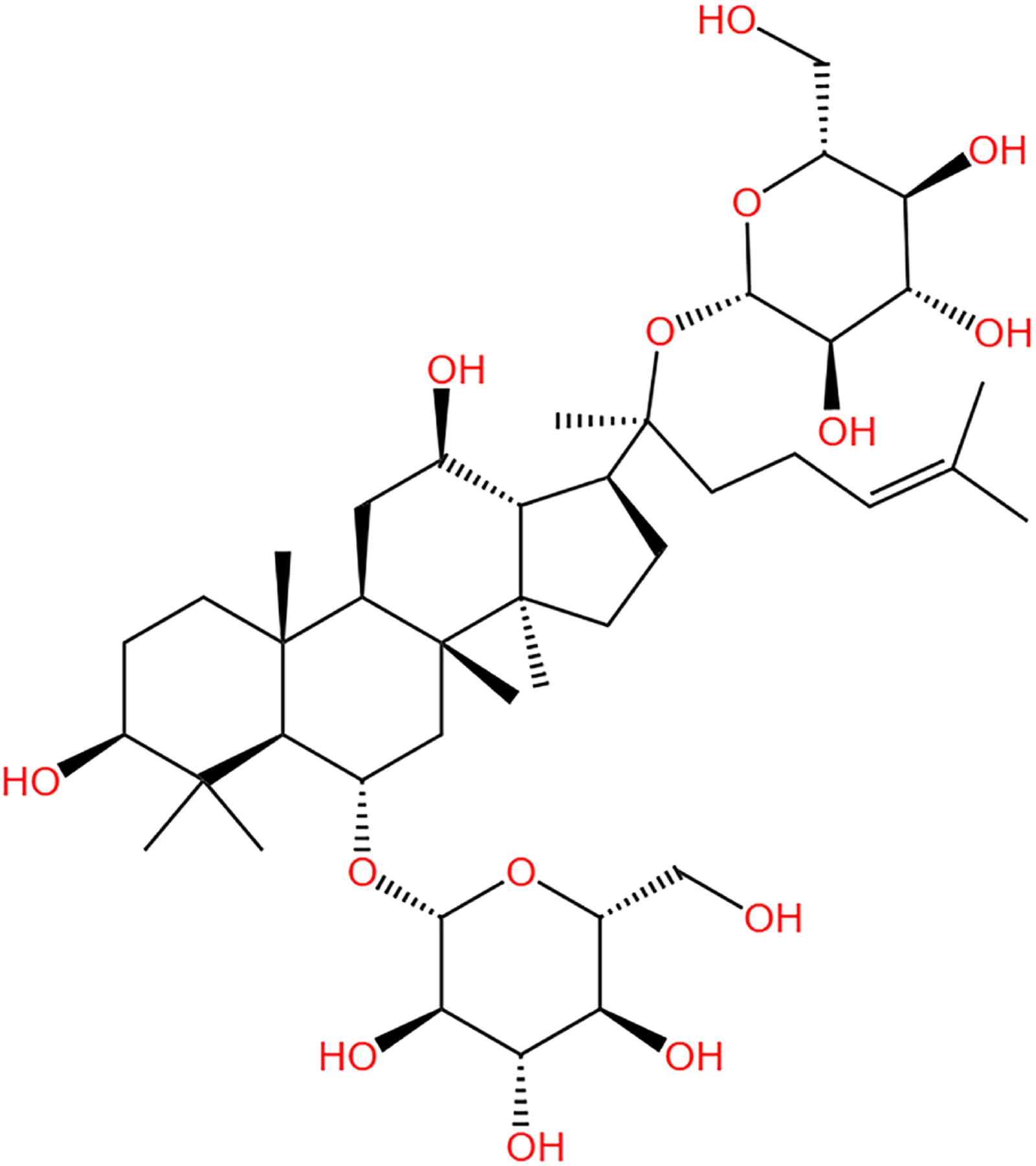
Chemical structures of ginsenoside Rg1.

## 2 Methods

### 2.1 Search strategies

A comprehensive literature search was conducted across four major biomedical databases (PubMed, EMBASE, Web of Science and the Cochrane Library) from inception to 31 May 2026. The core search strategy included the following concept-based keyword combinations: (“ginsenoside Rg1” OR “G-Rg1”) AND (“coronary artery ligation” OR "myocardial infarction" OR "ischemia-reperfusion" OR "ischemic heart disease" OR "myocardial injury"). In addition, the reference lists of potential relevant articles were also searched for relevant studies. All included studies were limited to animal experiments.

### 2.2 Eligibility criteria

Predefined inclusion criteria were as follows: (1) studies that used in vivo animal models of myocardial ischemia induced by either transient coronary artery occlusion followed by reperfusion or permanent ligation of the left anterior descending (LAD) coronary artery, and in which G-Rg1 was administered as an intervention; (2) studies that compared G-Rg1 monotherapy (any dose and route of administration) with a control group receiving vehicle (normal saline) or no treatment; (3) studies reporting at least one prespecified primary outcome: myocardial infarct size, plasma or serum levels of cardiac biomarkers (including CK-MB, lactate dehydrogenase [LDH], cardiac troponin I or T [cTnI/cTnT]), and left ventricular ejection fraction (LVEF). Secondary outcomes included mechanistic endpoints related to G-Rg1 on myocardial injury. Predefined exclusion criteria were as follows: (1) non-animal studies; (2) studies lacking a concurrent control group; (3) studies with missing, unreportable, or irretrievable outcome data essential for meta-analysis; and (4) duplicate publications or studies with overlapping datasets.

### 2.3 Data extraction

Data extraction was performed independently by two authors. The following information were systematically extracted: (1) first author name and publication year; (2) animal characteristics (species, strain, number of animals per group, sex and mean body weight); (3) myocardial injury models and anesthesia methods used for model preparation; (4) treatment group details, including dose, route of administration, treatment duration and control group information; (5) primary outcomes; and (6) summary statistics for each outcome, expressed as mean ± standard deviation (SD). In studies reporting multiple G-Rg1 doses, data from the highest administered dose were prioritized for the primary analysis to assess maximal efficacy under experimental conditions. For studies presenting quantitative results solely in graphical form, we contacted the corresponding authors via email to request raw or summary data.

### 2.4 Risk of bias in individual studies

Risk of bias was assessed using a slightly modified 10-point checklist [23] that comprised the following items: (A) peer-reviewed publication status; (B) maintenance of normothermia during surgery and recovery; (C) random allocation to treatment or control groups; (D) blinding during model induction; (E) blinding during outcome assessment; (F) use of an anesthetic agent with no documented direct cardioprotective activity; (G) use of clinically relevant comorbid animal models; (H) prospective justification of sample size; (I) adherence to national or institutional animal welfare guidelines; and (J) transparent disclosure of funding sources and potential conflicts of interest. Each item was scored one point. Two authors independently evaluated the quality of the studies, and the final results were determined through discussion to resolve any discrepancies.

### 2.5 Statistical analysis

Statistical heterogeneity across studies was formally assessed using the Cochran's Q test, with *P* < 0.05 considered statistically significant. The *I*^*2*^ statistic was also used to evaluate heterogeneity among subgroups. A fixed-effect model (*I*^*2*^ < 50%) or a random-effect model (*I*^*2*^ > 50%) was selected for meta-analysis based on the *I*^*2*^ value. The mean difference (MD) or standardized mean difference (SMD) and 95% confidence interval (CI) were used as effect sizes for combined analysis, and a funnel plot was drawn to assess publication bias. When high heterogeneity was present (*I*^*2*^ > 75%), subgroup analysis was conducted to identify the cause of high heterogeneity, or sensitivity analysis was performed by omitting one study at a time to verify the robustness of the main outcome. The combined analysis was completed using Revman 5.3 software. This study was a literature review and did not require ethical approval.

## 3 Results

### 3.1 Study selection

A total of 426 articles were identified through the literature search. After removing 218 duplicates, 208 unique records remained. Title and abstract screening excluded 176 records for the following prespecified reasons: (1) narrative reviews, systematic reviews, or editorials; (2) retracted publications, conference abstracts, letters, or protocols; and (3) studies unrelated to myocardial I/R injury or infarction. Ultimately, 9 studies met the selection criteria and were included in the analysis (Fig. 2).

**Fig. 2:**
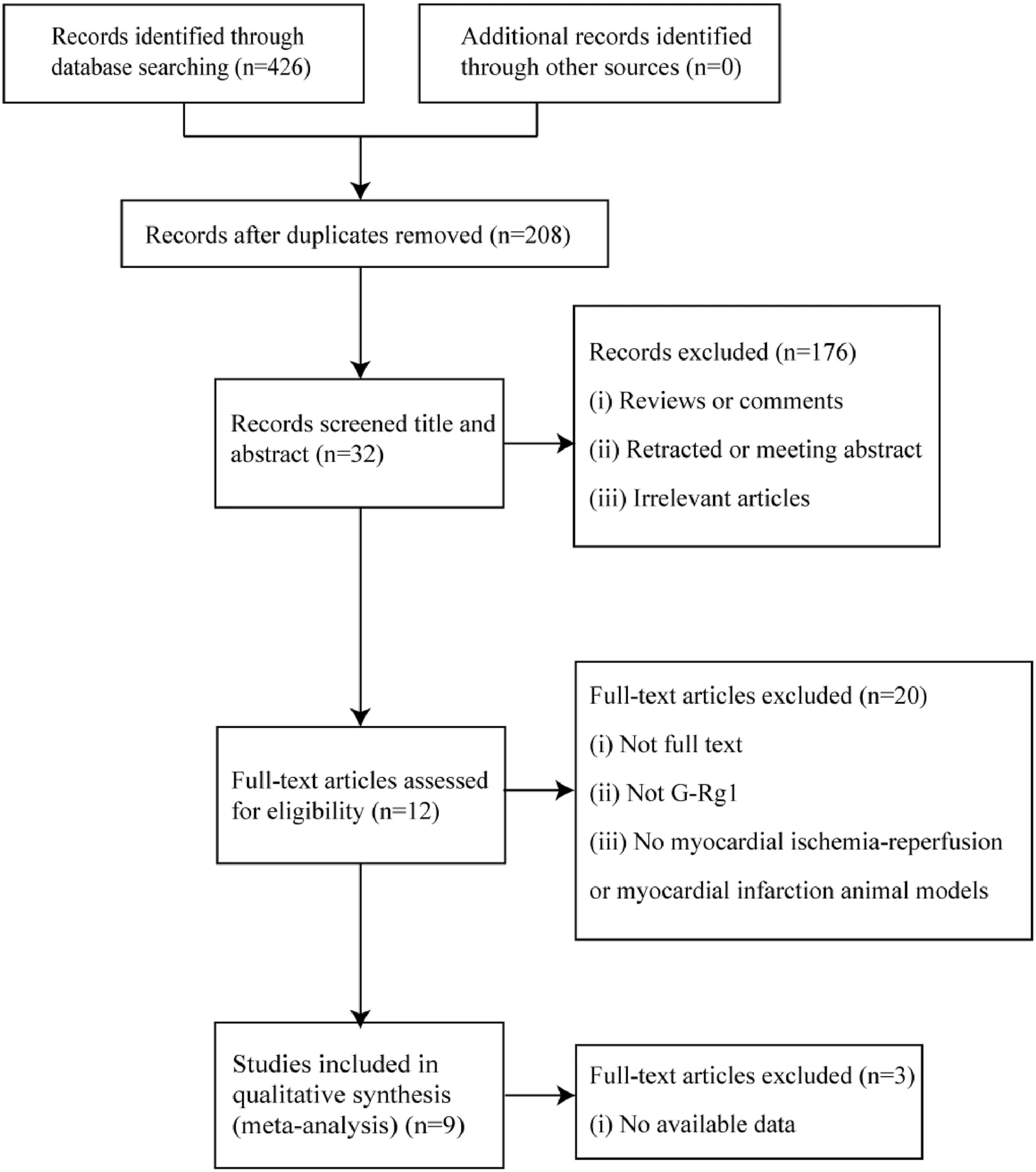
Summary of the process for screening candidate studies.

### 3.2 Characteristics included in the study

A total of 9 preclinical studies [[24], [25], [26], [27], [28], [29], [30], [31], [32]] were included, encompassing adult Sprague-Dawley rats, Wistar rats and C57BL/6 mice. Rat body weights ranged from 170 to 320 g, no body weight data were reported for mice. Seven studies [24,[27], [28], [29], [30], [31], [32]] exclusively used male animals, while two studies [25,26] employed both sexes (male and female rats). Anesthesia was induced with isoflurane in one study [27], chloral hydrate in one [32], and sodium pentobarbital in seven [[24], [25], [26],[28], [29], [30], [31]]. Myocardial injury was modeled via permanent LAD coronary artery ligation in three studies [[24], [25], [26]] and transient I/R in six [[27], [28], [29], [30], [31], [32]]. G-Rg1 was administered as a dose gradient in three studies [25,27,30] and at a fixed dose in six [24,26,28,29,31,32]. Primary outcomes varied across studies: myocardial infarct size was assessed in five studies [[25], [26], [27],^29^,^31^]; myocardial fibrosis in three [24,25,27]; LVEF and left ventricular fractional shortening (LVFS) in three [24,25,28]; and hemodynamic parameters (+dP/dt max and -dP/dt max) and myocardial blood flow (MBF) each reported in one study [29]. Regarding biochemical markers, LDH was reported in three studies [27,28,31]; CK-MB in two studies [27,28]; CK in one study [31]; apoptosis-associated speck-like protein (ASC) in two studies [27,28]; N-terminal pro-B-type natriuretic peptide (NT-pro BNP) in one study [25]; superoxide dismutase (SOD) and myocardial cell apoptosis rate in one study [26]; interleukin-1β (IL-1β) and tumor necrosis factor-α (TNF-α) in one study [27] NLR family pyrin domain-containing protein 3 (NLRP3) in one study [28]; Bax/Bcl-2 and cardiac cTnT in one study [29]; silent information regulator (SIRT), phosphatase and tensin homolog-induced kinase 1 (PINK1), E3 ubiquitin ligase (Parkin), microtubule-associated protein 1 light chain 3-II (LC3-II) and ubiquitin-binding protein P62 (P62) in one study [24]; inducibility of ventricular tachyarrhythmia in one study [30]; soluble vascular cell adhesion molecule-1 (sVCAM-1) in one study; and nitric oxide (NO) in one study [31]. The overall characteristics of the included publications are shown in Table 1.

**Table 1:** Summary of key characteristics of the 9 included studies.

Study (year)	Species (sex, n = experimental/control group)	Weight	Model (method)	Anesthetic	Treatment regimen	Control group	Outcome index (time)	Intergroup differences	Mechanism
Guan (2023) [24]	C57BL/6 mice (male, 8/8)	Not reported	LAD	Sodium pentobarbital (100 mg/kg)	Oral gavage administration of G-Rg1 (20 mg/kg) was initiated 6 h prior to surgery and continued once daily for 28 consecutive days	Oral gavage with 0.9% NaCl solution was initiated 6 h prior to surgery and continued once daily for 28 consecutive days	(1) LVEF (2) LVFS (3) Myocardial fibrosis (4) SIRT1 (5) PINK1 (6) Parkin (7) LC3-II (8) p62	(1) *P* < 0.01 (2) *P* < 0.01 (3) *P* < 0.05 (4) *P* < 0.01 (5) *P* < 0.01 (6) *P* < 0.01 (7) *P* < 0.05 (8) *P* < 0.05	SIRT1/PINK1/Parkin mitophagy signaling pathway
Xiang (2026) [25]	Wistar rats (male/female, 12/12)	180-220 g	LAD	Sodium pentobarbital (50 mg/kg)	G-Rg1 (40 mg/kg) was administered via intravenous infusion beginning on postoperative day 3 and continued once daily for seven consecutive days	No treatment	(1) LVEF (2) LVFS (3) NT-proBNP (4) Myocardial infarct size (5) Myocardial fibrosis	(1) *P* < 0.05 (2) *P* < 0.01 (3) *P* < 0.01 (4) *P* < 0.05 (5) *P* < 0.05	SH2B1- mediated inflammatory and fibrotic pathways
Yang (2022) [26]	Wistar rats (male/female, 10/10)	275-316 g	LAD	Sodium pentobarbital (40 mg/kg)	Intraperitoneal injection of G-Rg1 (40 mg/kg) was administered within 1 h after surgery and continued once daily for seven consecutive days	An equivalent volume of 0.9% NaCl solution was intraperitoneally injected within 1 h after surgery and once daily for seven consecutive days	(1) SOD (2) Myocardial infarct size (3) Myocardial cell apoptosis rate	(1) *P* < 0.05 (2) *P* < 0.05 (3) *P* < 0.05	CDK4/FGF9 signaling pathway and Bax/Bcl-2-mediated apoptotic pathway
Xu (2024) [27]	SD rats (male, 12/12)	250-300 g	I/R	3% isoflurane	G-Rg1 (20 mg/kg) was administered intravenously twice daily, once 2 h prior to LAD ligation and once immediately after reperfusion, with treatment initiated preoperatively and sustained for seven consecutive days	Distilled water (0.01 mL/g) was administered intravenously twice daily, once 2 h prior to LAD ligation and once immediately after reperfusion, for seven consecutive days	(1) Myocardial infarct size (2) CK-MB (3) LDH (4) IL-1β (5) TNF-α (6) ASC (7 )Myocardial fibrosis	(1) *P* < 0.01 (2) *P* < 0.01 (3) *P* < 0.01 (4) *P* < 0.01 (5) *P* < 0.01 (6) *P* < 0.01 (7) *P* < 0.01	AIM2 inflammasome -mediated macrophage M1/M2 polarization pathway
Li (2025) [28]	SD rats (male, 15/15)	210-260 g	I/R	Sodium pentobarbital (50 mg/kg)	G-Rg1 (40 mg/kg) was administered intraperitoneally once daily for seven days prior to model induction	Normal saline was administered intraperitoneally once daily for seven days prior to model induction	(1) CK-MB (2) LDH (3) LVEF (4) LVFS (5) NLRP3 (6) ASC	(1) *P* < 0.05 (2) *P* < 0.05 (3) *P* < 0.05 (4) *P* < 0.05 (5) *P* < 0.05 (6) *P* < 0.05	NLRP3/Caspase-1/GSDMD-mediated pyroptotic pathway
Li (2018) [29]	SD rats (male, 18/18)	240-260 g	I/R	Sodium pentobarbital (60 mg/kg)	G-Rg1 (5 mg/kg/h) was infused intravenously via a femoral vein catheter from 30 min before ischemia until the end of reperfusion (total 150 min)	Normal saline was infused intravenously via a femoral vein catheter from 30 min before ischemia until the end of reperfusion (total 150 min)	(1) Myocardial infarct size (2) MBF (3) cTnI (4) + dp/dt max (5) -dp/dt max (6) Bax/Bcl-2 (7) Complex I activity (8) Complex II activity (9) ATP synthase activity	(1) *P* < 0.05 (2 *)P* < 0.05 (3) *P* < 0.05 (4) *P* < 0.05 (5) *P* < 0.05 (6) *P* < 0.05 (7) *P* < 0.05 (8) *P* < 0.05 (9) *P* < 0.05	RhoA/ROCK mitochondrial energy metabolism pathway
Wang (2025) [30]	SD rats (male, 10/10)	170-230 g	Isolated heart I/R	Sodium pentobarbital (50 mg/kg)	G-Rg1 (40 mg/L) was dissolved in Tyrode's solution, and isolated hearts were perfused for 30 min before inducing I/R	Isolated hearts were continuously perfused with normoxic, non-ischemic Tyrode's solution	(1) Inducibility of ventricular (2) tachyarrhythmias (3) P-AKT (4) P-PKA (5) cAMP	(1) *P* < 0.05 (2) *P* < 0.05 (3) *P* < 0.05 (4) *P* < 0.05	L-type calcium channels-cAMP-PKA pathway and PI3K-AKT pathway
Qin (2018) [31]	SD rats (male, 8/8)	260-320 g	I/R	Sodium pentobarbital (40 mg/kg)	G-Rg1 (10 mg/kg) was administered via oral gavage 60 min prior to surgery	Normal saline was administered via oral gavage at an equivalent volume 60 min prior to surgery	(1) Myocardial infarct size (2) CK (3) LDH (4) NO	(1) *P* < 0.01 (2) *P* < 0.001 (3) *P* < 0.001 *(4)P* < 0.01	PI3K-AKT-mTOR signaling pathway
Deng (2015) [32]	Wistar rats (male, 25/25)	250-300 g	I/R	Chloral hydrate (330 mg/kg)	G-Rg1 (10 mg/kg) was given at the onset of reperfusion following ischemia	Normal saline was given at the onset of reperfusion following ischemia	(1) sVCAM-1	(1) *P* < 0.05	sVCAM-1

Footnote: LAD, left anterior descending coronary artery ligation; I/R, ischemia-reperfusion injury; Isolated heart I/R, isolated heart ischemia-reperfusion model; LVEF, left ventricular ejection fraction; LVFS, left ventricular fractional shortening; LVDP, left ventricular developed pressure; +dp/dt max, maximum rate of positive left ventricular pressure development; -dp/dt max, maximum rate of negative left ventricular pressure decline; MBF:myocardial blood flow; CK-MB, creatine kinase-MB isoenzyme; CK:creatine kinase; LDH, lactate dehydrogenase; cTnI, cardiac troponin I; NT-proBNP, N-terminal pro-B-type natriuretic peptide; SOD, superoxide dismutase; GSH-Px, glutathione peroxidase; NO, nitric oxide; sVCAM-1, soluble vascular cell adhesion molecule-1; IL-1β, interleukin-1β; TNF-α, tumor necrosis factor-α; ASC, apoptosis-associated speck-like protein containing a CARD; AIM2, absent in melanoma 2; NLRP3, NOD-like receptor family pyrin domain-containing 3; Caspase-1, cysteinyl aspartate specific proteinase-1; GSDMD, gasdermin D; SIRT1, silent information regulator 1; PINK1, PTEN-induced putative kinase 1; Parkin, parkin RBR E3 ubiquitin protein ligase; LC3-II, microtubule-associated protein 1 light chain 3-II; p62, sequestosome 1; Bax, Bcl-2-associated X protein; Bcl-2, B-cell lymphoma-2; Complex I/II, mitochondrial respiratory chain complex I/II; CDK4, cyclin-dependent kinase 4; FGF9, fibroblast growth factor 9; SH2B1, SH2 domain-containing adapter protein B1; RhoA, Ras homolog family member A; ROCK, Rho-associated coiled-coil containing protein kinase; cAMP, cyclic adenosine monophosphate; PKA, protein kinase A; P-PKA, phosphorylated protein kinase A; PI3K, phosphatidylinositol 3-kinase; P-AKT, phosphorylated protein kinase B; G-Rg1, ginsenoside Rg1; mg/kg, in vivo administration dosage for intact animals; mg/L, G-Rg1 concentration dissolved in Tyrode's solution for isolated heart perfusion; *P*, statistical significance value.

### 3.3 Study quality

The methodological quality scores of the included studies ranged from 4 to 6 out of a maximum of 10. All studies were published in peer-reviewed journals, and animals were randomly allocated to experimental groups. Six studies [25,[28], [29], [30], [31], [32]] reported control of ambient temperature. All nine studies employed anesthetics that lacked significant intrinsic vascular protective effects. No study reported sample size calculation, blinding during model induction, or blinding during outcome assessment. All nine studies complied with established animal welfare regulations, and seven studies [[24], [25], [26], [27], [28],^30^,^31^] explicitly declared no competing interests. A summary of the methodological quality is provided in Table 2.

**Table 2:** Characteristics of the 9 included studies.

Study (year)	A	B	C	D	E	F	G	H	I	J	Total
Guan (2023) [24]	✓		✓			✓			✓	✓	5
Xiang (2026) [25]	✓	✓	✓			✓				✓	5
Yang (2022) [26]	✓		✓			✓				✓	4
Xu (2024) [27]	✓		✓			✓			✓	✓	5
Li (2025) [28]	✓	✓	✓			✓			✓	✓	6
Li (2018) [29]	✓	✓	✓			✓			✓		5
Wang (2025) [30]	✓	✓	✓			✓			✓	✓	6
Qin (2018) [31]	✓	✓	✓			✓			✓	✓	6
Deng (2015) [32]	✓	✓	✓			✓			✓		5

Studies fulfilling the following methodological criteria: (A) publication in a peer-reviewed journal; (B) control of ambient temperature; (C) random allocation to treatment or control groups; (D) blinding during model induction; (E) blinding during outcome assessment; (F) use of anesthetics lacking significant intrinsic vascular protective effects; (G) use of physiologically relevant animal models (e.g., aged, diabetic, or hypertensive); (H) a priori sample size calculation; (I) adherence to established animal welfare regulations; (J) explicit declaration of competing interests.

## 4 Effectiveness

### 4.1 Myocardial infarct size and myocardial fibrosis

A meta-analysis of five studies [[25], [26], [27],^29^,^31^] demonstrated that G-Rg1 significantly reduced myocardial infarct size in animal models of ischemia-induced myocardial injury relative to controls (n = 28; SMD = -1.73, 95% CI: -2.42 to -1.03; *P* < 0.00001; heterogeneity: χ2 = 4.31, df = 4, P = 0.37, *I*^*2*^ = 7%) (Fig. 3).

**Fig. 3:**
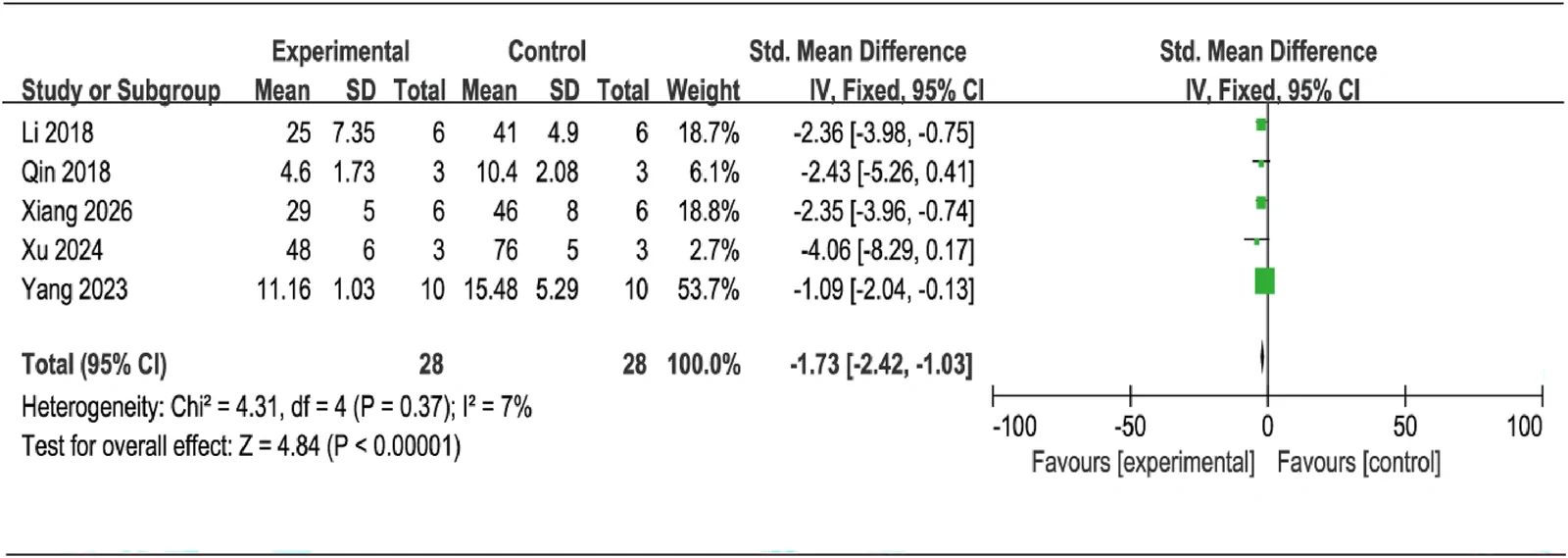
The forest plot: effects of G-Rg1 for decreasing the MI size compared with control group.

A meta-analysis of three studies [24,25,27], encompassing 50 experimental animals (25 in the G-Rg1 group and 25 in the control group), assessed the effect of G-Rg1 on myocardial fibrosis in animal models of ischemia-induced myocardial injury. Significant heterogeneity was observed across studies (χ^2^ = 8.07, df = 2, P = 0.02; *I*^*2*^ = 75%) (Fig. 4); consequently, a random-effects model was applied (τ^2^ = 2.57) (Fig. 4). The observed heterogeneity (*I*^*2*^ = 75%) was moderate and mainly originated from differences in animal models, intervention protocols, and fibrosis measurement methods across the included studies. Despite this heterogeneity, all studies reported effect estimates in the same direction (reduction in fibrosis), supporting the robustness of the pooled estimate as reflecting genuine biological efficacy rather than methodological artifact. The overall effect size was substantial and statistically significant (SMD = -3.61, 95% CI: -5.72 to -1.51; *P* = 0.0008) (Fig. 4), indicating that G-Rg1 significantly attenuates myocardial fibrosis in ischemia-injured animal models relative to controls.

**Fig. 4:**
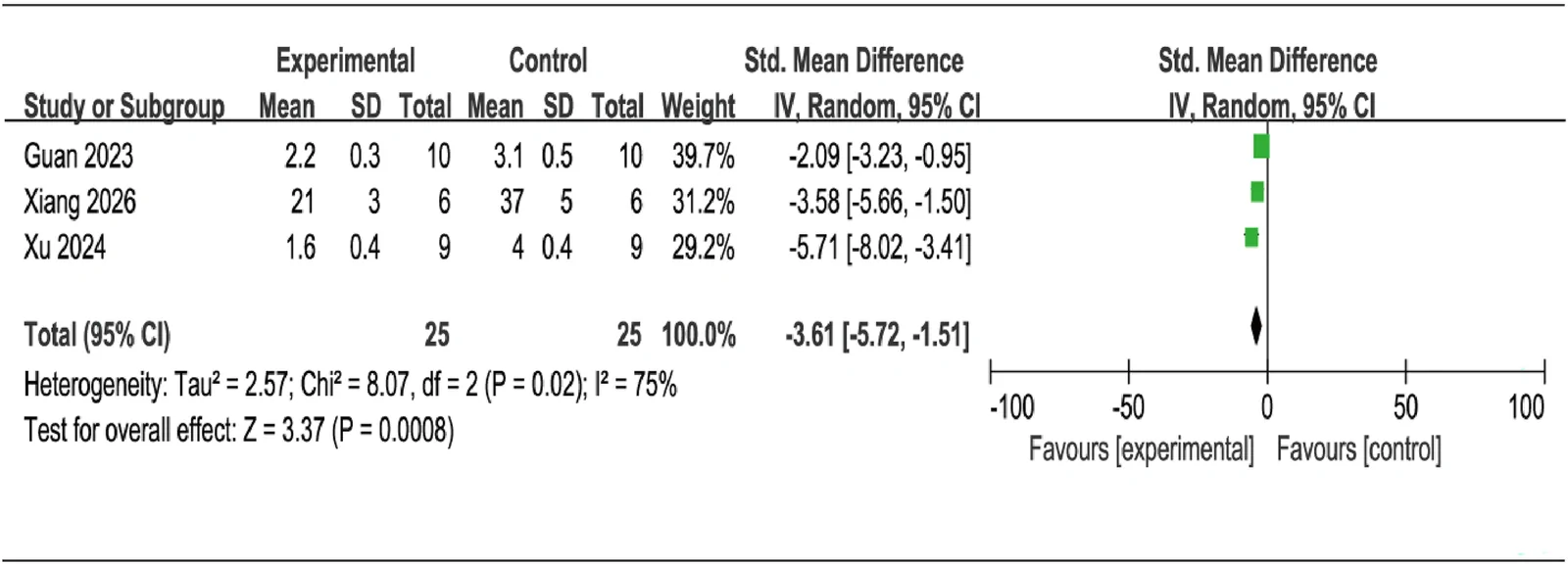
The forest plot: effects of G-Rg1 for decreasing the myocardial fibrosis compared with control group.

### 4.2 CK-MB, LDH, CK, NT-pro BNP, cTnI

A meta-analysis of two studies [27,28], involving a total of 30 animals (15 in the G-Rg1 group and 15 in the control group), assessed the effect of G-Rg1 on serum CK-MB levels in animal models of ischemia-induced myocardial injury. Substantial heterogeneity was observed (χ^2^ = 5.33, df = 1, *P* = 0.02; *I*^*2*^ = 81%) (Fig. 5), therefore, a random-effects model was applied (τ^2^ = 5.13) (Fig. 5). The analysis indicated that the main source of heterogeneity was differences in animal model construction, intervention protocols, and outcome measurement methods among the included studies. Moreover, all included studies showed a consistent effect direction, suggesting that the heterogeneity reflected reasonable methodological and clinical heterogeneity. The overall pooled results demonstrated that G-Rg1 significantly reduced serum CK-MB levels in ischemia-induced myocardial injury compared with the control group (SMD = -4.75, 95% CI: -8.38 to -1.12, *P* = 0.01) (Fig. 5).

**Fig. 5:**
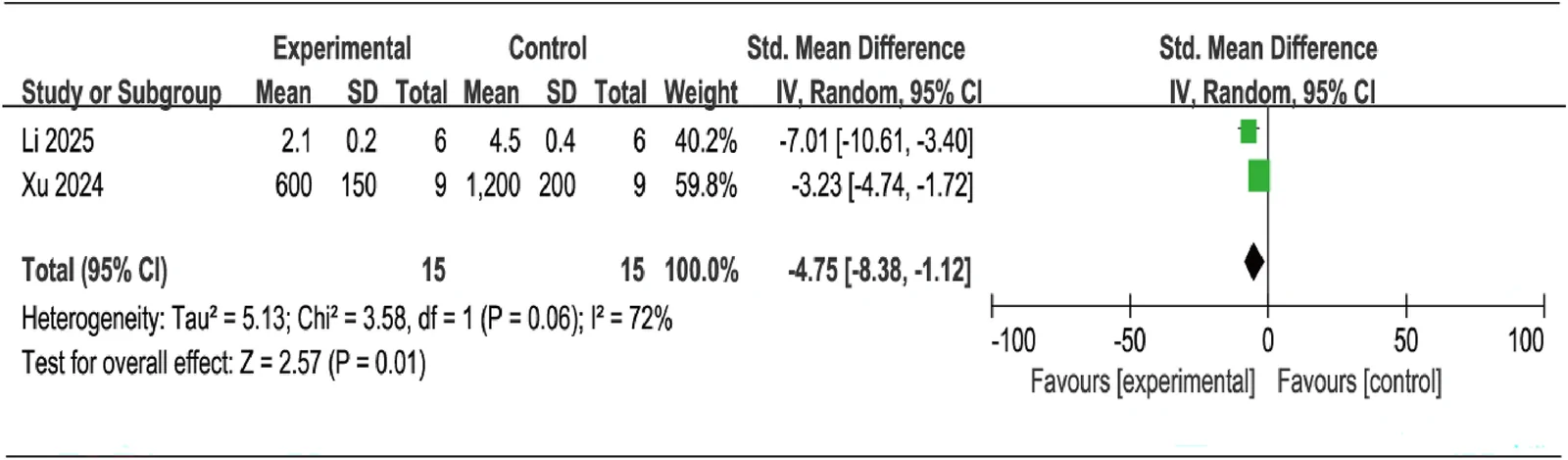
The forest plot: effects of G-Rg1 for decreasing CK-MB Isoenzyme compared with control group.

A meta-analysis of three studies [27,28,31], encompassing 46 experimental animals (23 in the G-Rg1 group and 23 in the control group), evaluated the effect of G-Rg1 on serum LDH activity in animal models of ischemia-induced myocardial injury. Significant heterogeneity was observed across studies (χ^2^ = 8.10, df = 2, *P* = 0.02; *I*^*2*^ = 75%) (Fig. 6); thus, a random-effects model was applied (τ^2^ = 4.02) (Fig. 6). The moderate heterogeneity was mainly attributable to differences in intervention protocols and measurement methods, and all included studies the same effect direction, indicating that the heterogeneity represented reasonable clinical heterogeneity. The pooled estimate demonstrated a large and highly significant reduction in LDH (SMD = -4.79, 95% CI: -7.55 to -2.03; *P* = 0.0007) (Fig. 6), confirming that G-Rg1 significantly attenuates myocardial cellular injury as indexed by LDH release in ischemia-injured animal models relative to controls.

**Fig. 6:**
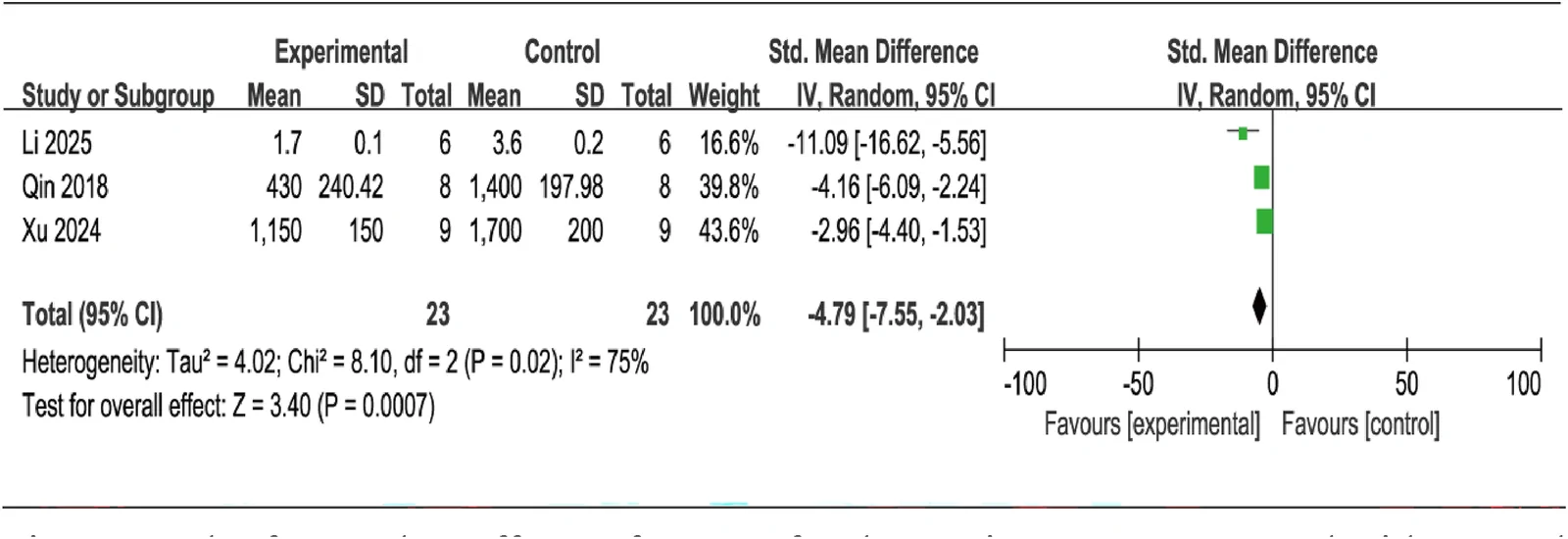
The forest plot: effects of G-Rg1 for decreasing LDH compared with control group.

One study [31] reported a statistically significant reduction in serum CK levels following G-Rg1 treatment compared with the control group (*P* < 0.001); one study [25] observed a significant decrease in NT-pro BNP concentrations with G-Rg1 relative to the control (*P* < 0.01); and one study [29] documented a significant attenuation of cTnI release after G-Rg1 administration compared with control (*P* < 0.05).

### 4.3 LVEF and LVFS

A meta-analysis of three studies [24,25,28] demonstrated that G-Rg1 significantly improved LVEF in animal models of ischemia-induced myocardial injury compared with controls (n = 28; SMD = 4.20, 95% CI: 3.16-5.24; *P* < 0.00001; heterogeneity: χ^2^ = 3.33, df = 2, *P* = 0.19, *I*^*2*^ = 40%) (Fig. 7). Similarly, a meta-analysis of the same three studies [24,25,28] showed that G-Rg1 significant improved LVFS compared with controls (n = 28; SMD = 3.96, 95% CI: 2.98-4.95; *P* < 0.00001; heterogeneity: χ^2^ = 0.51, df = 2, *P* = 0.78, *I*^*2*^ = 0%) (Fig. 8).

**Fig. 7:**
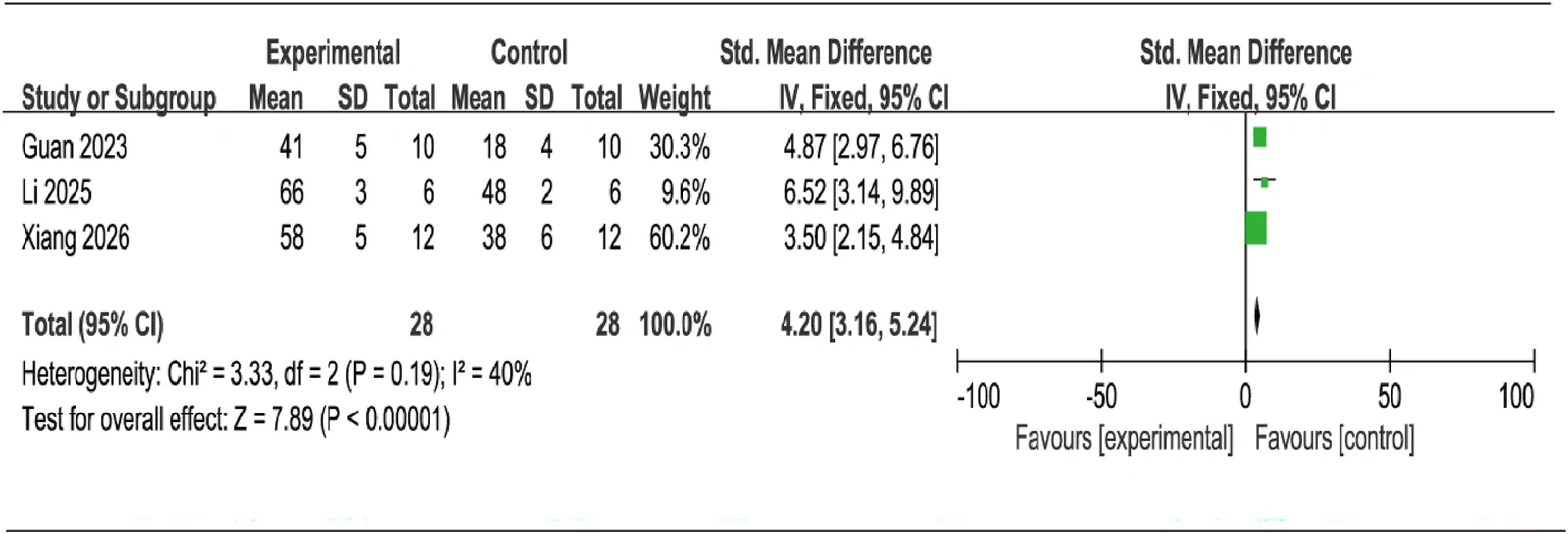
The forest plot: effects of G-Rg1 for increasing LVEF compared with control group.

**Fig. 8:**
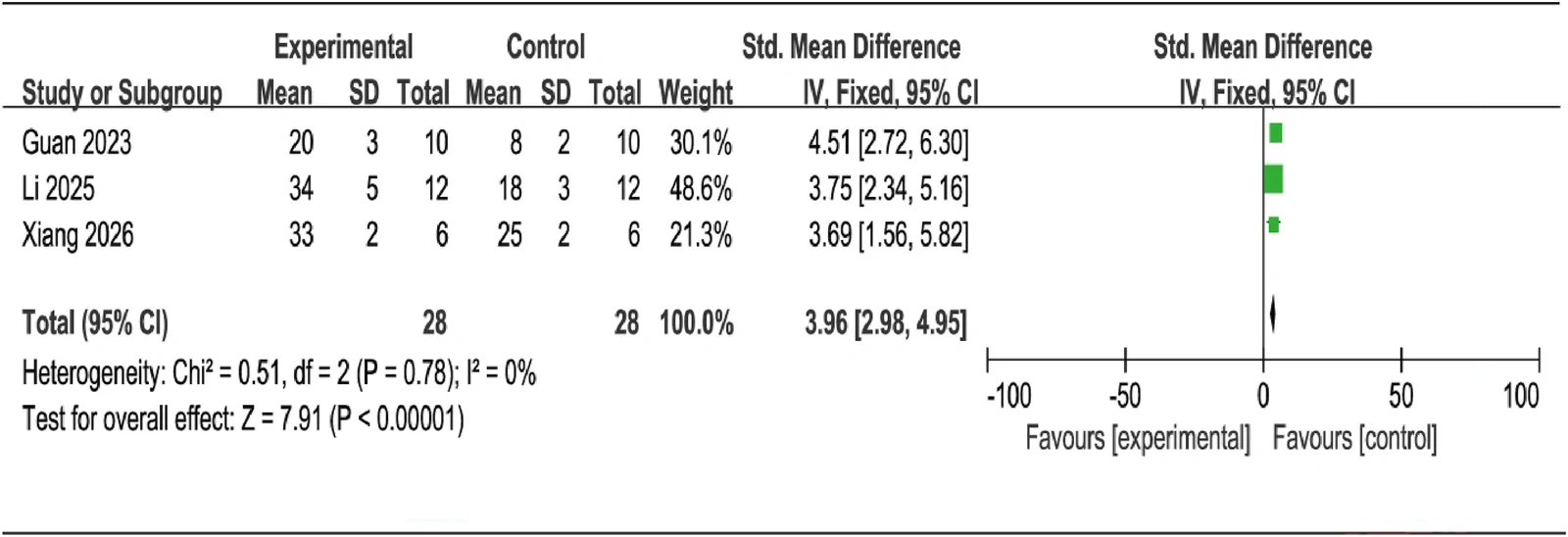
The forest plot: effects of G-Rg1 for increasing LVFS compared with control group.

### 4.4 MBF, inducibility of ventricular tachyarrhythmias, +dP/dt max and -dP/dt max

One study [29] reported that G-Rg1 significantly increased MBF relative to control (*P* < 0.05). Another study [30] demonstrated that G-Rg1 significantly reduced the inducibility of ventricular tachyarrhythmias compared with control (*P* < 0.05). A third study [29] further showed that G-Rg1 significantly improved the maximum rates of left ventricular pressure rise (+dP/dt) and decline (−dP/dt) (*P* < 0.05).

## 5 Cardioprotective effect

### 5.1 Anti-apoptotic and anti-pyroptotic

Compared with the control group, a meta-analysis of two independent studies [27,28] demonstrated that G-Rg1 significantly attenuated the expression of ASC in ischemia-induced myocardial injury (n = 12; SMD = -5.04; 95% CI: -6.96 to -3.11; χ^2^ = 0.05, df = 1, *P* = 0.82; *I*^*2*^ = 0%) (Fig. 9). Additional supporting evidence from individual studies indicates that G-Rg1 consistently modulated key molecular effectors of programmed cell death: it reduced the Bax/Bcl-2 ratio [29] (*P* < 0.05), decreased myocardial cell apoptosis rate [26] (*P* < 0.05), suppressed cleavage of Caspase-1 [28] (*P* < 0.05) and Caspase-3 [29] (*P* < 0.05), lowered the cleaved Caspase-1/Caspase-1 ratio [27] (*P* < 0.01), diminished P62 accumulation [24] (*P* < 0.05), and inhibited proteolytic activation of gasdermin D (cleaved GSDMD) [28] (*P* < 0.05). Collectively, these findings suggest that G-Rg1 exerts multifaceted anti-apoptotic and anti-pyroptotic effects in the ischemic myocardium.

**Fig. 9:**
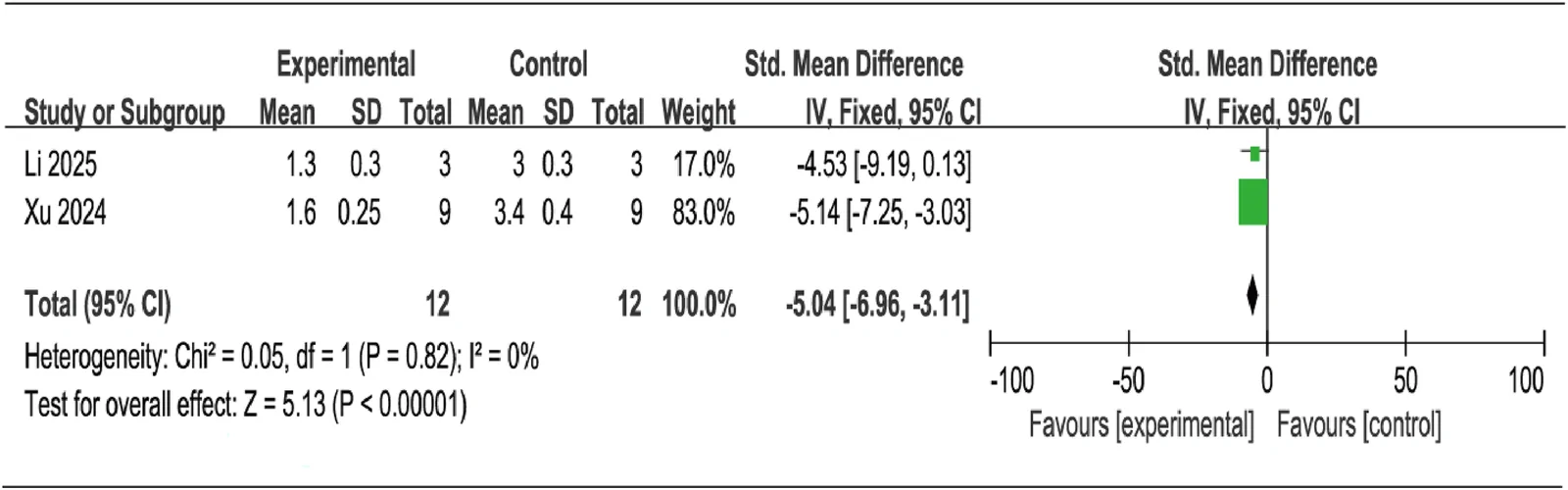
The forest plot: effects of G-Rg1 for decreasing ASC compared with control group.

### 5.2 Suppress inflammation

A single study [27] reported significant reductions in the pro-inflammatory cytokines TNF-α (*P* < 0.01), AIM2 (*P* < 0.01) and IL-1β (*P* < 0.01). Another study [28] demonstrated inhibition of NLRP3 inflammasome activation. A third study [32] showed decreased sVCAM-1 levels (*P* < 0.05).

### 5.3 Mitochondrial protective effect

A study [24] demonstrated that G-Rg1 activated the SIRT1/PINK1/Parkin signaling axis (*P* < 0.01), upregulated LC3-II protein levels (*P* < 0.05), and downregulated P62/SQSTM1 expression (*P* < 0.05). These findings are consistent with enhanced mitophagy. Another study [29] reported that G-Rg1 significantly improved the enzymatic activities of mitochondrial respiratory chain complexes I and II, as well as ATP synthase (all *P* < 0.05), leading to increased cellular ATP production (*P* < 0.05).

### 5.4 Other mechanisms

A study [26] reported significantly increased SOD activity (*P* < 0.05). Another study [31] demonstrated elevated NO levels (*P* < 0.01).

## 6 Discussion

### 6.1 Summary of evidence

This preclinical systematic review evaluated the efficacy of G-Rg1 against ischemia-induced myocardial injury. Nine studies comprising 236 animals met the inclusion criteria, and the overall methodological quality was rated moderate. The findings indicate that G-Rg1 confers cardioprotection primarily through three interrelated mechanisms: attenuation of inflammation, suppression of cardiomyocyte apoptosis, and preservation of mitochondrial structure and function.

### 6.2 Comparative pharmacological profile of G-Rg1

Why has G-Rg1 gained far more research focus for treating ischemic myocardial injury? Compared with other components derived from ginseng, G-Rg1 possesses multiple unique pharmacological properties. First, G-Rg1 displays a more comprehensive targeting capacity in regulating pathways related to myocardial injury, allowing simultaneous intervention in key pathological processes such as mitochondrial homeostasis, antioxidant stress, immune regulation, calcium electrophysiological balance, apoptosis, myocardial fibrosis and endoplasmic reticulum (ER) stress. Specifically, G-Rg1 can coordinately modulate multiple core mechanisms of ischemic injury, including SIRT1/PINK1/Parkin-mediated mitophagy, RhoA/Rho-associated coiled-coil containing protein kinase (ROCK)-mediated mitochondrial energy metabolism, nuclear factor erythroid 2-related factor 2 (Nrf2)-mediated antioxidant response, NLRP3/AIM2 inflammasome-driven pyroptosis, the SH2B1/NF-κB immune regulatory axis, L-type calcium channel-mediated calcium overload, PI3K/AKT-mediated cell survival, PERK/CHOP-mediated ER stress and fibrosis-related pathways [20,21,[24], [25], [26], [27], [28], [29], [30], [31], [32], [33], [34], [35], [36], [37], [38]]. In contrast, the mechanisms of other major ginsenoside monomers are relatively limited: G-Rb1 primarily targets AMPK-mediated mitophagy and basal NF-κB inflammatory regulation, and no clear evidence yet demonstrates its direct involvement in calcium homeostasis or ER stress [[39], [40], [41], [42], [43]]; research on ginsenoside Rg3 (G-Rg3) mainly focuses on cardiac hypertrophy and glucolipid metabolism regulation, lacking systematic mechanistic exploration of calcium channel function, antiarrhythmic effects and angiogenesis [[44], [45], [46], [47]]; ginsenoside Re (G-Re) exerts mild anti-inflammatory and antifibrotic effects predominantly through estrogen receptor signaling and a single microRNA pathway [48,49]; ginsenoside Rc (G-Rc) is confined to Nrf2-dependent antioxidant activity and basic SIRT1-mediated anti-apoptotic effects [[50], [51], [52], [53], [54]], failing to integrate key pathological nodes such as calcium overload and mitochondrial dynamics imbalance, thus limiting its capacity to achieve multidimensional intervention throughout the entire course of myocardial injury. Notably, only in the case of G-Rg1 has the PI3K/AKT pathway, a central hub in the aforementioned network, been shown to mediate cross-talk across multiple pathways, such as mitochondrial repair, calcium homeostasis maintenance, anti-inflammatory effects, and anti-fibrotic actions. In contrast, other saponin monomers can only activate AKT in isolation and lack the capacity for cross-pathway synergistic regulation. Second, G-Rg1 is applicable to a broader range of myocardial injury models and is supported by more substantial research evidence, particularly showing significantly greater depth and breadth in elucidating the mechanisms of myocardial I/R injury compared with other saponin monomers. Third, G-Rg1 exhibits bidirectional dynamic regulation of autophagy, capable of appropriately upregulating or suppressing autophagic activity depending on the type and stage of injury, whereas G-Rb1 only displays unidirectional promotion of mitophagy. Finally, G-Rg1, a protopanaxatriol-type saponin, exhibits better water solubility than the protopanaxadiol-type G-Rg3. In contrast, G-Rb1 has poor lipid solubility, resulting in low passive transmembrane efficiency. Consequently, G-Rg1 demonstrates significantly superior oral absorption and myocardial targeting accumulation compared with G-Rg3 and G-Rb1. Moreover, G-Rg1 is supported by more extensive evidence from both in vitro and in vivo mechanistic studies, showing protective effects against various cardiovascular injuries, including ischemia, oxidative stress, inflammation and drug toxicity. In summary, G-Rg1 can systematically intervene in the key stages of IHD across its entire course, encompassing acute-phase myocardial necrosis, intermediate-phase electrophysiological disturbances and inflammatory responses, and long-term ventricular remodeling. Its comprehensive protective efficacy and clinical translational potential are markedly superior to those of other known major ginsenosides. Key details are presented in Table 3.

**Table 3:** Summary of molecular targets, mechanisms of action, and cardiovascular effects of major ginsenosides.

Ginsenoside	Molecular targets	Mechanism	Cardioprotective effects	References
G-Rg1	Nrf2 and JNK	Nrf2/HO-1 signaling and JNK signaling	G-Rg1 attenuates H/R-induced oxidative stress injury in cardiomyocytes by activating the Nrf2/HO-1 signaling pathway and inhibiting JNK phosphorylation.	Li (2017) [20]
	Glutamate dehydrogenase and mitofusin 2	Mitochondrial dynamics balance	G-Rg1 effectively preserves mitochondrial dynamics homeostasis in H/R-injured cardiomyocytes.	Dong (2016) [21]
	SIRT1	SIRT1/PINK1/Parkin mitophagy pathway	G-Rg1 activates SIRT1-mediated mitophagy, inhibiting ventricular remodeling following myocardial infarction.	Guan (2023) [24]
	SH2B1	SH2B1-mediated inflammatory and fibrotic signaling pathways.	G-Rg1 attenuates myocardial interstitial fibrosis by inhibiting fibroblast activation and decreasing collagen deposition through downregulating SH2B1 expression.	Xiang (2026) [25]
	CDK4 and Bax	CDK4/FGF9 signaling pathway and Bax/Bcl-2-mediated apoptotic pathway	G-Rg1 promotes cardiomyocyte survival by upregulating the CDK4/FGF9 signaling pathway, modulates the Bax/Bcl-2 ratio to inhibit mitochondrial apoptosis, and synergistically enhances neovascularization in the infarct area, ultimately reducing myocardial infarct size.	Yang (2022) [26]
	AIM2	AIM2 inflammasome-mediated macrophage M1/M2 polarization pathway	G-Rg1 inhibits AIM2 inflammasome activation, promotes macrophage polarization from a pro-inflammatory M1 to a reparative M2 phenotype, and attenuates myocardial inflammatory infiltration and fibrotic injury.	Xu (2024) [27]
	NLRP3	NLRP3/Caspase-1/GSDMD-mediated pyroptotic pathway	G-Rg1 suppresses NLRP3 inflammasome activation, inhibits cardiomyocyte pyroptosis, enhances the activities of the antioxidant enzymes SOD and glutathione peroxidase (GSH-Px), and reduces oxidative stress and myocardial necrosis markers.	Li (2025) [28]
	RhoA	RhoA/ROCK mitochondrial energy metabolism pathway	G-Rg1 inhibits the RhoA/ROCK pathway, restores mitochondrial respiratory chain function, enhances ATP synthesis, ameliorates energy metabolic disturbances in the ischemic myocardium, and preserves myocardial contractile integrity.	Li (2018) [29]
	L-type calcium channels	L-type calcium channels-cAMP-PKA pathway and PI3K-AKT pathway	G-Rg1 attenuates calcium overload in cardiomyocytes by inhibiting L-type calcium channel-mediated calcium influx while concurrently activating the PI3K-AKT signaling pathway, thereby improving electrophysiological homeostasis and markedly reducing the incidence of malignant arrhythmias induced by ischemia/reperfusion.	Wang (2025) [30]
	PI3K	PI3K-AKT-mTOR signaling pathway	G-Rg1 suppresses pathological autophagy by activating the PI3K-AKT-mTOR signaling pathway and upregulates HIF-1α expression to promote angiogenesis, thereby attenuating hypoxia-induced cardiomyocyte necrosis.	Qin (2018) [31]
	sVCAM-1	Inflammatory response	G-Rg1 attenuates inflammatory responses and I/R-induced myocardial injury and fibrosis.	Deng (2015) [32]
	AMPKα	AMPK-mTOR signaling pathway	G-Rg1 inhibits AMPKα activation, promotes mTOR activation, and attenuates H/R-induced autophagy.	Zhang (2012) [33]
	transcriptional intermediary factor	Endoplasmic reticulum stress and autophagy	G-Rg1 alleviates endoplasmic reticulum stress, suppresses excessive autophagy downstream of the ER stress response, and attenuates doxorubicin-induced myocardial injury.	Xu (2018) [34]
	P2X7 receptor	AKT/GSK-3β signaling pathway	G-Rg1 activates the AKT/GSK-3β pathway via the P2X7 receptor, thereby suppressing sepsis-induced cardiac dysfunction and mitochondrial impairment.	Liu (2022) [35]
	AMPK and PERK	AMPK/mTOR signaling pathway and PERK/ATF4/CHOP signaling pathway	G-Rg1 attenuates alcohol-induced cardiomyocyte injury by suppressing autophagy and endoplasmic reticulum stress through inhibition of the AMPK/mTOR and PERK/ATF4/CHOP pathways.	Tian (2023) [36]
	ERK1/2	ERK1/2 signaling pathway	G-Rg1 ameliorates heart failure by downregulating ERK1/2 phosphorylation.	Peng (2024) [37]
	TLR4	TLR4/NF-κB/NLRP3 signaling pathway	G-Rg1 ameliorates sepsis-induced myocardial dysfunction in mice by inhibiting the TLR4/NF-κB/NLRP3 signaling pathway.	Luo (2020) [38]
G-Rb1	AMPK	AMPK signaling pathway	G-Rb1 promotes autophagy and enhances the survival of hypoxic cardiomyocytes by upregulating the AMPK signaling pathway.	Dai (2019) [39]
	AMPKα	Mitophagy	G-Rb1 protects against acute myocardial ischemic injury by promoting mitophagy through AMPKα phosphorylation.	Hu (2022) [40]
	FADD	PPARα signaling pathway	G-Rb1 inhibits FADD-mediated activation of the PPARα signaling pathway, thereby ameliorating ISO-induced heart failure.	Li (2023) [41]
	MicroRNA-21	Myocardial fibrosis	G-Rb1 downregulates exosomal microRNA-21 expression, attenuating myocardial fibrosis.	Wang (2026) [42]
	IL-6	Inflammatory response	G-Rb1 attenuates Ang II-induced cardiac hypertrophy in mice by suppressing inflammatory responses.	Wang (2021) [43]
G-Rg3	SIRT1	SIRT1/NF-κB signaling pathway	G-Rg3 attenuates Ang II-induced myocardial hypertrophy by inhibiting the NLRP3 inflammasome and suppressing oxidative stress through modulating the SIRT1/NF-κB pathway.	Ren (2021) [44]
	AMPK	AMPK signaling pathway	G-Rg3 ameliorates insulin resistance in mice with TAC-induced cardiac hypertrophy by activating the AMPK signaling pathway.	Ni (2022) [45]
	PPAR-γ	Adiponectin pathway	G-Rg3 activates the adiponectin pathway by binding to PPAR-γ, thereby regulating glucose and lipid metabolism to protect against diabetic cardiomyopathy.	Zhang (2023) [46]
	SUMO1	SUMOylation pathway of SERCA2a	G-Rg3 promotes SERCA2a SUMOylation to ameliorate cardiac dysfunction in heart failure.	Liu (2021) [47]
G-Re	Estrogen receptors	PI3K/AKT signaling pathway	G-Re attenuates lipopolysaccharide-induced cardiac dysfunction and inflammatory responses.	Chen (2016) [48]
	MicroRNA-489	MicroRNA-489/MyD88/NF-κB signaling pathway	G-Re promotes microRNA-489 transcription, suppresses MyD88 expression and NF-κB phosphorylation, thereby inhibiting Ang II-induced myocardial fibrosis.	Sun (2023) [49]
G-Rc	Nrf2	Oxidative stress and inflammatory response	G-Rc attenuates myocardial ischemic injury by upregulating Nrf2-mediated antioxidant and anti-inflammatory responses.	Shi (2022) [50]
	APN	AdipoR1/APPL1/LKB1/AMPKα2 signaling pathway	G-Rc attenuates lipotoxicity by promoting APN secretion and activating the AdipoR1/APPL1/LKB1/AMPKα2 signaling pathway, thereby preventing and ameliorating diabetic cardiomyopathy.	Han (2026) [51]
	STAT3	STAT3/FoxO3a/SIRT1 signaling pathway	G-Rc attenuates septic cardiomyopathy by suppressing STAT3/FoxO3a pathway protein expression, upregulating SIRT1, and modulating macrophage polarization.	Wang (2025) [52]
	SIRT1	SIRT1 signaling pathway	G-Rc attenuates myocardial injury induced by acute cold exposure in rats by modulating SIRT1 expression and reducing inflammatory responses.	Xue (2021) [53]
	SIRT1	SIRT1 signaling pathway	G-Rc attenuates myocardial ischemia-reperfusion injury by activating SIRT1, reducing mitochondrial oxidative stress and inhibiting apoptosis.	Xue (2023) [54]

Footnote: G-Rg1/G-Rb1/G-Rg3/G-Re/G-Rc, unified abbreviations of corresponding ginsenosides; Nrf2, nuclear factor erythroid 2-related factor 2; JNK, c-Jun N-terminal kinase; HO-1, heme oxygenase-1; SIRT1, silent information regulator 1; PINK1, PTEN-induced putative kinase 1; Parkin, parkin RBR E3 ubiquitin protein ligase; SH2B1, SH2 domain-containing adapter protein B1; CDK4, cyclin-dependent kinase 4; FGF9, fibroblast growth factor 9; Bax, Bcl-2-associated X protein; Bcl-2, B-cell lymphoma-2; AIM2, absent in melanoma 2; NLRP3, NOD-like receptor family pyrin domain-containing 3; Caspase-1, cysteinyl aspartate specific proteinase-1; GSDMD, gasdermin D; SOD, superoxide dismutase; GSH-Px, glutathione peroxidase; RhoA, Ras homolog family member A; ROCK, Rho-associated coiled-coil containing protein kinase; cAMP, cyclic adenosine monophosphate; PKA, protein kinase A; PI3K, phosphatidylinositol 3-kinase; AKT, protein kinase B; mTOR, mammalian target of rapamycin; HIF-1α, hypoxia-inducible factor-1α; sVCAM-1, soluble vascular cell adhesion molecule-1; AMPKα, AMP-activated protein kinase α subunit; P2X7, P2X purinoceptor 7; GSK-3β, glycogen synthase kinase 3β; PERK, protein kinase R-like endoplasmic reticulum kinase; ATF4, activating transcription factor 4; CHOP, C/EBP homologous protein; ERK1/2, extracellular regulated protein kinases 1/2; TLR4, toll-like receptor 4; NF-κB, nuclear factor κB; FADD, Fas-associated death domain; PPARα, peroxisome proliferator-activated receptor α; ISO, isoproterenol; MicroRNA-21, miR-21; IL-6, interleukin-6; Ang II, angiotensin II; PPAR-γ, peroxisome proliferator-activated receptor γ; SUMO1, small ubiquitin-like modifier 1; SERCA2a, sarcoplasmic/endoplasmic reticulum calcium ATPase 2a; MicroRNA-489, miR-489; MyD88, myeloid differentiation primary response gene 88; APN, adiponectin; AdipoR1, adiponectin receptor 1; APPL1, adaptor protein containing PH domain, PTB domain and leucine zipper motif 1; LKB1, liver kinase B1; AMPKα2, AMP-activated protein kinase α2 subunit; STAT3, signal transducer and activator of transcription 3; FoxO3a, forkhead box O3a; H/R, hypoxia/reoxygenation; I/R, ischemia-reperfusion; TAC, transverse aortic constriction; M1, pro-inflammatory macrophage phenotype; M2, reparative macrophage phenotype.

### 6.3 An integrated signaling network for G-Rg1 cardioprotection

This study integrates findings from nine relevant publications to constructs an integrated signaling pathway (Fig. 10), confirming that myocardial I/R initiates a self-amplifying pathological cascade. I/R injury begins with mitochondrial dysfunction and massive release of mitochondrial reactive oxygen species (mtROS). The released mtROS acts as common second messengers, simultaneously triggering oxidative stress, NF-κB/NLRP3/AIM2-mediated inflammation, and calcium overload caused by excessive activation of L-type calcium channels, thereby setting off multiple mutually reinforcing vicious cycles. Mitochondrial damage results in excessive opening of the mitochondrial permeability transition pore, which in turn exacerbates calcium overload, while oxidative stress and inflammatory factors further promote mtROS release.

**Fig. 10:**
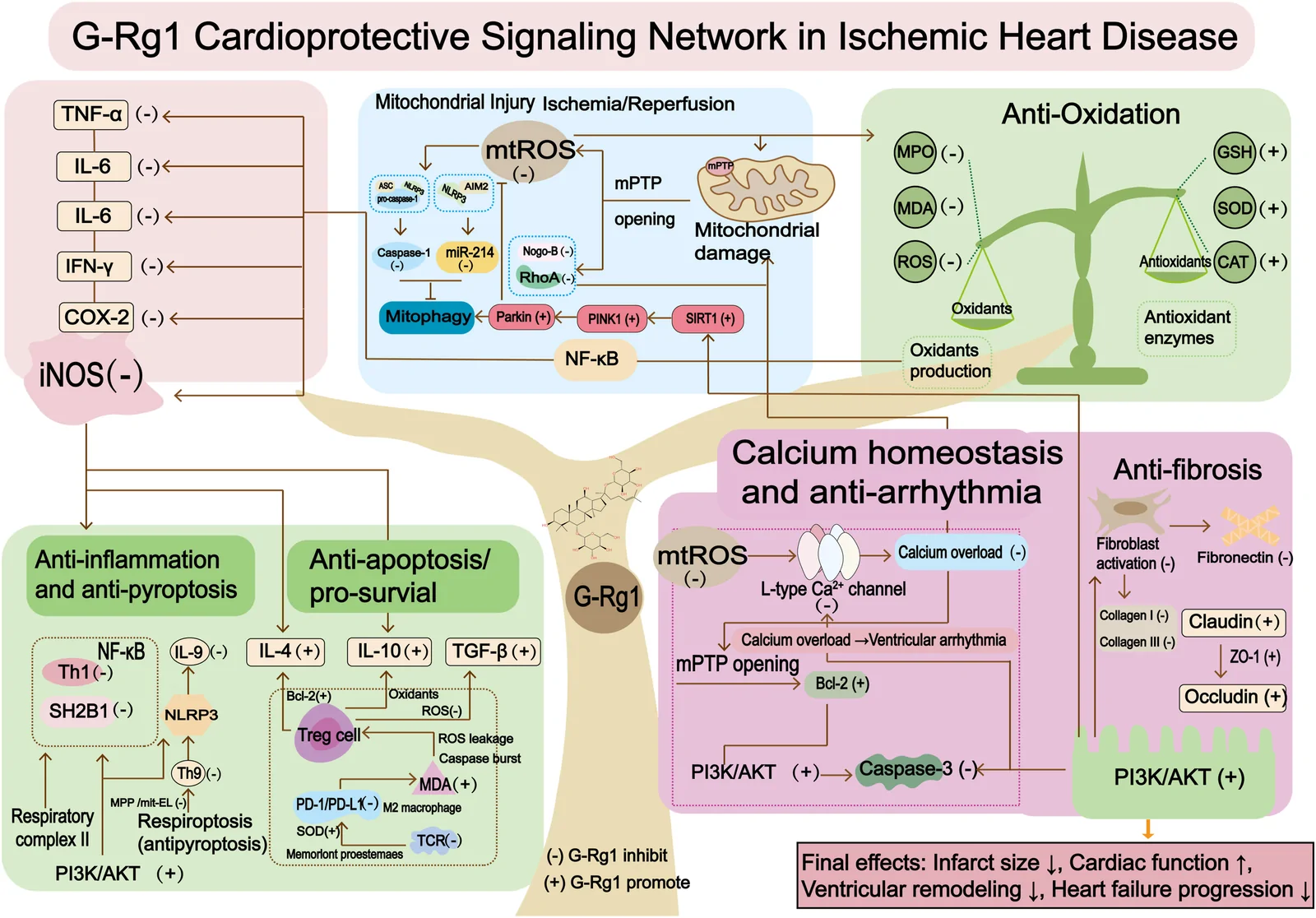
G-Rg1 cardioprotective signaling network in ischemic heart disease.

G-Rg1 protects against I/R-induced myocardial injury not through a single signaling pathway but via multiple targets and pathways. Among these, the SIRT1/PINK1/Parkin mitophagy pathway and the PI3K/AKT pro-survival pathway function as two core synergistic hubs. At the upstream level, G-Rg1 activates SIRT1-mediated mitophagy to remove dysfunctional mitochondria and suppress mtROS overproduction, while simultaneously inhibiting the RhoA/Nogo-B pathway to stabilize mitochondrial structure, thereby blocking downstream pathological stimuli. Concurrently, G-Rg1 enhances the endogenous antioxidant system, scavenges excessive reactive oxygen species, and disrupts the positive feedback loop between oxidative stress and inflammation. Regarding immune homeostasis, G-Rg1 exerts bidirectional regulation on two immune pathways. On one hand, it blocks the SH2B1/NF-κB-Th1/Th9 pro-inflammatory cascade and suppresses pyroptosis driven by NLRP3/AIM2 inflammasomes. On the other hand, it promotes Treg cell expansion and induces the secretion of IL-4, IL-10 and TGF-β, thereby establishing a negative feedback loop that alleviates inflammatory injury. The PI3K/AKT pathway serves as a transmembrane central hub, integrating multiple protective effects, including calcium electrophysiological protection, anti-apoptotic actions, mitochondrial repair, and anti-fibrotic effects. Specifically, it inhibits excessive calcium influx to relieve calcium overload, blocks Caspase-3-mediated cardiomyocyte death, enhances SIRT1-dependent mitophagy through a positive feedback mechanism, and directly suppresses fibroblast activation, thereby reducing the synthesis of fibronectin and type I/III collagen. Long-term fibrotic ventricular remodeling represents the terminal pathological outcome of cumulative oxidative injury, inflammation, and apoptosis. G-Rg1 restores the expression of myocardial tight junction proteins such as zonula occludens-1, claudin, and occludin, limiting the spread of injury signals within the myocardial interstitium and further attenuating the progression of fibrosis.

In summary, the signaling pathways regulated by G-Rg1 exhibit extensive hierarchical interactions rather than functioning independently. G-Rg1 establishes an integrated and unified regulatory network that may provide cardioprotection across different stages of ischemic injury, from acute infarct injury to long-term cardiac remodeling, ultimately resulting in reduced myocardial infarct area, improved cardiac contractile and diastolic function, alleviated pathological ventricular remodeling, and slowed progression of heart failure.

### 6.4 Translational challenges and a clinical development roadmap

Despite reproducible preclinical efficacy, the clinical translation of G-Rg1 remains impeded by several translational barriers. Oral bioavailability and pharmacokinetics: the oral bioavailability of G-Rg1 in rats ranges from approximately 1.9% to 20.0% of the administered dose [55], owing mainly to its high polarity, poor membrane permeability, and active biliary excretion. Following intravenous administration, the α-phase half-life is 24 min, indicating a rapid decline in effective plasma concentrations after peak levels are reached. Despite a β-phase half-life of 14.13 h, sustaining therapeutic plasma concentrations remains challenging [56]. Clinically achievable concentrations: when dosing is adjusted by body surface area, the effective dose in rodents such as mice ranges from 5 to 40 mg/kg, corresponding to a human equivalent dose of 0.55 to 6.35 mg/kg. Literature data indicate that co-administration of *Salvia miltiorrhiza* B and G-Rg1 at a 2:5 ratio and a combined dose of 10 mg/kg yields an intravenous median lethal dose (LD50) in mice of 1747 mg/kg. From this, the acute intravenous LD50 of G-Rg1 alone can be calculated as approximately 1248 mg/kg [57]. Although this value is markedly higher than the effective dose and suggests a wide safety margin, it does not necessarily reflect long-term oral safety. Long-term safety: Although the acute toxicity of G-Rg1 is very low, caution is warranted regarding hormone-sensitive tissues during prolonged treatment because of its potent phytoestrogenic activity. For instance, intravenous administration of G-Rg1 (6 mg/kg/day) for four consecutive days significantly increased apoptosis in blastocyst-stage embryos in mice and negatively affected early embryonic development [58]. Additionally, potential interactions between G-Rg1 and other drugs require further investigation.

As detailed below, all available preclinical data have been derived from young, healthy animal models lacking the comorbidities typical of patients with IHD. To facilitate clinical translation, we propose the following strategy: (I) develop G-Rg1 ester prodrugs, encapsulate G-Rg1 within phospholipid bilayers to form complexes, or explore alternative approaches, such as inhibiting efflux transporters and modifying administration routes (sublingual or transdermal delivery), to enhance oral bioavailability and prolong systemic exposure; (II) conduct efficacy validation in large-animal models with comorbidities, including diabetes, hypertension, atrial fibrillation, renal insufficiency, and/or advanced age; (III) evaluate target engagement and safety using human iPSC-derived cardiomyocytes and cardiac organoids.

### 6.5 Limitations

First, our search was restricted to English-language publications, which may have led to a certain degree of selection bias [59]. Only nine studies comprising 236 animals met the inclusion criteria. The small number of included studies reduced the statistical power for detecting publication bias. Second, the underreporting of negative or null results, common in preclinical research, may contribute to an upward bias in the estimated magnitude of G-Rg1's cardioprotective effect [60]. Third, the methodological quality of the included studies was assessed as moderate (scores ranging from 4 to 6 on a standardized scale). In the relevant literature, none of the studies reported an a priori sample size calculation, and most did not implement researcher blinding during the surgical model induction. These omissions substantially increase the risk of performance and detection bias, warranting cautious interpretation of pooled conclusions [61]. Furthermore, ischemia-induced myocardial injury in humans frequently occurs against a background of comorbidities, including aging, diabetes mellitus, hypertension, and dyslipidemia [62], yet none of the included animal studies incorporated models reflecting these clinically relevant metabolic or age-related conditions. The absence of these clinically relevant conditions severely limits the generalizability of preclinical findings. Finally, sex-specific biological factors represent an additional limitation: two studies [25,26] used female animals, but did not control for estrogen-mediated cardioprotection, which may have affected the observed therapeutic effects [63].

Given these limitations, we strongly recommend that future preclinical studies adhere to the Animal Research: Reporting of In Vivo Experiments (ARRIVE 2.0 guidelines), with particular emphasis on preregistration, blinded randomization, appropriate a priori sample size justification, and inclusion of animal models with comorbidities. Rigorous clinical trials should also be conducted to validate the efficacy and safety of G-Rg1, thereby paving the way for its integration into therapeutic strategies for IHD.

### 6.6 Summary and outlook

The heart is a high-energy-demand organ whose sustained contractile function critically depends on mitochondrial oxidative phosphorylation. In IHD, mitochondria serve not only as the central hub of myocardial energy metabolism but also as pivotal regulators of cardiomyocyte death and endogenous cardioprotection [64]. Consequently, mitochondrial pathways are increasingly recognized as promising therapeutic targets in IHD [[65], [66], [67]]. In summary, the available evidence indicates that G-Rg1 confers cardioprotection partly by preserving mitochondrial integrity and bioenergetic capacity through activation of the SIRT1/PINK1/Parkin axis, leading to enhanced stability and activity of mitochondrial respiratory complexes I and II as well as ATP synthase. Moreover, G-Rg1 significantly reduces infarct size, attenuates interstitial fibrosis, and improves left ventricular systolic and diastolic function. Collectively, our findings indicate that G-Rg1 mediates myocardial protection in IHD via three synergistic mechanisms: suppression of inflammation, inhibition of apoptosis, and preservation of mitochondrial structure and function.

However, positive intervention effects observed in preclinical animal studies frequently fail to translate to human clinical trials, reflecting both low translational efficiency and poor consistency of results [68]. Inadequate reporting, particularly of methodological details, severely impedes therapeutic development, while irreproducible findings undermine the credibility of preclinical evidence and hinder its progression into novel, clinically viable treatments for human disease [68,69]. High-quality randomized controlled trials (RCTs) remain the gold standard for evaluating the efficacy and safety of medical interventions [70]. Rigorously designed and faithfully conducted RCTs yield the highest level of evidence for intervention effects and meaningfully improve patient-centered outcomes [71]. All nine included studies employed random allocation during group assignment. Randomization strengthens internal validity by minimizing imbalances in known and unknown confounders across treatment arms. Across the research continuum from basic discovery and translational investigation to clinical validation, animal models serve as indispensable tools for modeling human pathophysiology and informing therapeutic strategies [72]. Preclinical animal studies are essential for elucidating disease mechanisms, identifying and validating novel drug targets, and characterizing pharmacokinetic, toxicological, and drug-drug interaction profiles [73]. To address widespread deficiencies in reporting transparency and methodological rigor, the ARRIVE 2.0 guidelines were introduced in 2020 [74], which list the items that any in vivo experimental paper should include to ensure comprehensive and transparent description. Given that incomplete methodological reporting remains pervasive in preclinical literature, we strongly recommend that future studies adhere strictly to ARRIVE 2.0 guidelines, particularly in justifying sample size, implementing investigator blinding, and ensuring outcome assessor blinding, to enhance reliability, reduce bias, and strengthen translational relevance.

Therefore, the clinical translation potential of G-Rg1 remains unclear until obstacles such as oral bioavailability, model generalizability, and methodological reproducibility are addressed. G-Rg1 should be viewed not as a candidate for immediate clinical trials, but rather as a high-priority molecular prototype whose further development requires rigorous preclinical validation under clinically relevant conditions.

## 7 Conclusion

In recent years, a growing body of preclinical evidence has highlighted the therapeutic potential of ginsenosides, particularly the prototypical monomer G-Rg1, in CVD. This review summarizes the preclinical cardioprotective effects of G-Rg1 on ischemic myocardial injury and its potential molecular mechanisms, aiming to provide new insights and a theoretical basis for preventing and treating IHD. Our systematic review indicates that G-Rg1 alleviates myocardial injury in preclinical models by reducing infarct size, suppressing interstitial fibrosis, improving contractile function, promoting angiogenesis in the peri-infarct region, and preserving mitochondrial structural and functional integrity. Mechanistically, the cardioprotective effects of G-Rg1 are mediated through a coordinated signaling network that integrates anti-inflammatory, anti-apoptotic, and mitochondrial-protective pathways, with evidence suggesting that the SIRT1/PINK1/Parkin, PI3K/AKT/mTOR, and inflammasome regulatory axes serve as the core components. Compared to other major ginsenosides, G-Rg1 exhibits unique pharmacological properties, including broader therapeutic applicability, more comprehensive signaling pathway regulation, bidirectional regulation of autophagy, and superior oral absorption and myocardial targeted enrichment.

Although preclinical evidence substantiates the cardioprotective properties of G-Rg1, current research is limited by the small number of studies, moderate methodological quality, the lack of animal models with comorbidities, and significant pharmacokinetic barriers. Future research should focus on developing high-bioavailability formulations and delivery methods, conducting rigorous validation in large animal models with comorbid conditions, and identifying the direct molecular targets of G-Rg1. Rigorously designed prospective clinical trials are essential to validate mechanistic findings from bench studies and determine whether G-Rg1 holds genuine therapeutic promise for IHD. These studies should examine dosage, long-term efficacy, and safety to fully determine the clinical potential of G-Rg1. Furthermore, while current research has identified multiple molecular targets of G-Rg1 in ischemic myocardium, the hierarchical organization, crosstalk, and context-dependent regulation within its signaling network remain incompletely resolved. Integrative multi-omics approaches, including quantitative proteomics, spatial metabolomics, and CRISPR-based functional genomics offer unprecedented opportunities to yield new insights into the multi-dimensional network of G-Rg1.

In summary, these preclinical findings support future translational research and lay the foundation for subsequent clinical studies evaluating the therapeutic potential of G-Rg1 in myocardial injury.

## Author contributions

Jia-xin Chen: Writing-original draft, Data curation, Conceptualization. San-jian Hu and Yu-jie Zhang: Data curation. Zi-you Liu and Yu-ting Huang: Writing-review & editing, Supervision, Project administration, Funding acquisition, Conceptualization.

## Conflicts of interest

The authors declare no conflicts of interest.

## Abbreviations:

CVD: cardiovascular disease
IHD: ischemic heart disease
I/R: ischemia/reperfusion
MI: myocardial infarction
G-Rg1: ginsenoside Rg1
LAD: left anterior descending
CK-MB: creatine kinase-MB
LDH: lactate dehydrogenase
cTnI/cTnT: cardiac troponin I or T
LVEF: left ventricular ejection fraction
LVFS: left ventricular fractional shortening
MBF: myocardial blood flow
ASC: apoptosis-associated speck-like protein
NT-pro BNP: N-terminal pro-B-type natriuretic peptide;
SOD: superoxide dismutase; IL-1β, interleukin-1β
TNF-α,: tumor necrosis factor-α;
NLRP3: NLR family pyrin domain-containing protein 3
SIRT: silent information regulator
PINK1: phosphatase and tensin homolog-induced kinase 1
Parkin: E3 ubiquitin ligase
LC3-II: microtubule-associated protein 1 light chain 3-II
P62: ubiquitin-binding protein P62
sVCAM-1: soluble vascular cell adhesion molecule-1
NO: nitric oxide;
AIM2: absent in melanoma 2
SH2B1: SH2 domain-containing adaptor protein 1
CDK4: cyclin-dependent kinase 4
FGF9: fibroblast growth factor 9
mtROS: mitochondrial reactive oxygen species
α-SMA: α-smooth muscle actin
GSH-Px: glutathione peroxidase
M1: M1-type macrophage
M2: M2-type macrophage
Caspase-1: cysteine-aspartic protease-1
GSDMD: gasdermin D
IL-18: interleukin-18
RhoA: ras homolog family member A
ICa,L,: L-type calcium current
PKA: protein kinase A
PI3K: phosphatidylinositol-3-kinase
mTOR: mammalian target of rapamycin
HIF-1α: hypoxia-inducible factor-1 α
CRISPR: clustered regularly interspaced short palindromic repeats.
